# PD90 Financial Evaluation For The Acquisition Of A Healthcare Technology In The Hospital Context: An Application Case

**DOI:** 10.1017/S0266462325102171

**Published:** 2025-12-29

**Authors:** Luis Esteban Orozco

## Abstract

**Introduction:**

Hospital-based health technology assessment is relevant for optimal decision-making, particularly in a low-middle income country like Colombia. As a vital tool for application at the regional level, we present the manual for studies in the hospital context of the Institute for Health Technology Assessment, through the applied case of the financial analysis of a hospital procurement process.

**Methods:**

A technology assessment was carried out from a hospital perspective to analyze the purchase of 12 medical devices in some hospitals in Colombia. The perspective was from the hospital and the data used were obtained from a review of the effectiveness and safety literature, from the hospital’s own information, and from consultation with the supplier. A financial analysis was carried out considering the main variables of relevance in relation to the costs of infrastructure, human capital, adaptation of the institution, and maintenance of the device. Likewise, a technical feasibility analysis of the implementation of the technology was carried out.

**Results:**

Through analysis, it was possible to recommend the feasibility of and the most efficient purchase options for the analyzed medical devices: three types of X-ray machines, two types of computed tomography scanners, an anesthesia machine, a bronchoscope, a bronchoscopy tower, an echocardiograph, a cardiac stress test, an ultrasound machine, and a vascular Doppler device. It was concluded that, in some cases, the technical analysis of implementation feasibility offers relevant information for the selection of investment in a health technology, and that this is additional to the financial analysis and the analysis of effectiveness and safety.

**Conclusions:**

We constructed a manual for evaluating hospital technologies in Colombia as well as an analysis of the acquisition of seven medical devices for some hospitals. This study provided the main guidelines for carrying out this type of analysis for the country and provided the tools for decision-makers to choose the most efficient investment.

